# Xiao’er Fengreqing oral liquid versus Xiao’er Yanbian granules for pediatric acute pharyngitis/tonsillitis (external wind heat syndrome): protocol and statistical analysis plan for a multi-center, randomized, double-blind, active drug-controlled trial

**DOI:** 10.3389/fphar.2025.1625547

**Published:** 2025-07-18

**Authors:** Kai Liu, Lei Shi, Yi-Ke Song, Yu Du, Yi Yuan, Ze-Yang Shi, Jian-Ping Liu, Hui-Lan Liu, Zhao-Lan Liu

**Affiliations:** ^1^ Centre for Evidence-Based Chinese Medicine, Beijing University of Chinese Medicine, Beijing, China; ^2^ Business Analytics, University of Colorado Denver, Denver, CO, United States; ^3^ The Third Affiliated Hospital of Beijing University of Chinese Medicine, Beijing, China

**Keywords:** pharyngitis, tonsillitis, Xiao’er Fengreqing oral Liquid, randomized controlled trial, non-inferiority design, trial protocol, statistical analysis plan

## Abstract

**Objective:**

To evaluate the effectiveness and safety of Xiao’er Fengreqing Oral Liquid (XFOL) for pediatric acute pharyngitis/tonsillitis (external wind heat syndrome) through a multi-center, randomized, double-blind, positive-controlled, non-inferiority clinical trial.

**Method:**

A total of 120 participants (60 per group) will be randomized to receive either XFOL or Xiao’er Yanbian Granules (positive control) for 5 days. The primary outcome is the throat pain resolution rate and overall effective rate at Day 5, assessed via the Wong-Baker Faces Pain Rating Scale (WBS). Secondary outcomes include time to symptom onset/resolution, fever resolution time, and traditional Chinese medicine (TCM) syndrome scores. Safety assessments will monitor adverse events, vital signs, and laboratory parameters. Statistical analyses will follow a pre-specified plan, employing non-inferiority testing, survival analysis for time-to-event endpoints, and generalized estimating equations for repeated measures. Missing data will be handled using the last observation carried forward (LOCF) method for effectiveness endpoints, while safety analyses will rely on observed cases.

**Conclusion:**

This trial will provide rigorous evidence on the non-inferiority and safety profile of Fengreqing Oral Liquid, supporting its integration into pediatric care for acute upper respiratory infections. Adherence to a predefined statistical analysis plan ensures transparency and minimizes bias, ultimately guiding evidence-based clinical practice for TCM interventions.

## Introduction

Pediatric acute pharyngitis/tonsillitis (PAPT) is an inflammatory condition of the upper respiratory tract, primarily affecting the pharyngeal mucosa and tonsils, with symptoms including sore throat, fever, dysphagia, and cervical lymphadenopathy (Van Driel et al., 2021). Viral pathogens account for 70%–80% of cases, while Group A *Streptococcus* (GAS) is the predominant bacterial cause, responsible for 15%–30% of cases in children aged 5–15 years (Le Marechal et al., 2013). Globally, PAPT represents a major pediatric health burden, contributing significantly to outpatient visits and antibiotic prescriptions (Carapetis et al., 2005). In high-income countries, the annual incidence ranges from 60 to 90 per 1,000 children, peaking during colder months (Spinks et al., 2021). In the U.S., cross-sectional studies have demonstrated that both acute and chronic tonsillitis contribute significantly to pediatric healthcare burden, with chronic cases requiring more frequent office visits and higher prescription medication use compared to acute cases (Duarte et al., 2015).

Current management of PAPT prioritizes accurate diagnosis to avoid unnecessary antibiotic use while preventing complications such as rheumatic fever. International guidelines, including those from the Infectious Diseases Society of America (IDSA) and the American Academy of Pediatrics (AAP), recommend rapid antigen detection tests (RADTs) or throat cultures to confirm GAS infection before initiating antibiotics (Wald et al., 2013; Shulman et al., 2012). First-line therapy for confirmed GAS pharyngitis remains oral penicillin or amoxicillin due to their narrow spectrum and cost-effectiveness (Van Driel et al., 2021). However, antibiotic overprescription persists globally, with studies reporting 60%–70% of viral cases receiving antimicrobials, exacerbating resistance rates for *Streptococcus* pneumoniae and *Haemophilus* influenzae (Klein et al., 2024). Macrolides and cephalosporins are reserved for penicillin-allergic patients, though emerging resistance to these agents has been documented in Asia and Europe (Klein, 1997). Nonsteroidal anti-inflammatory drugs (NSAIDs) and acetaminophen are widely used for symptomatic relief, but their overuse in children raises concerns about renal and hepatic toxicity (Fashner et al., 2012). Novel strategies, including GAS vaccines targeting M-protein epitopes and phage therapy (Wang et al., 2018), are under investigation but face challenges in effectiveness and scalability.

Emerging evidence supports the role of traditional Chinese medicine (TCM) in managing PAPT, particularly for viral or mild bacterial cases. Xiao’er Fengreqing Oral Liquid (XFOL), a 20-herb formulation including Lonicerae Japonicae Flos (honeysuckle), Forsythiae Fructus (forsythia), and Menthae Haplocalycis Herba (mint), among others, has demonstrated anti-inflammatory, antiviral, and immunomodulatory properties (Zhou et al., 2020). Produced by Handan Pharmaceutical Co., Ltd. (Handan, China), XFOL has been approved by the National Medical Products Administration of China for marketing (Z19990012) in 1995. A multicenter randomized controlled trial (RCT) comparing XFOL with Xiao’er Baotaikang Granules (control) showed comparable effectiveness in fever resolution (median time: 46 h vs. 46 h, P = 0.83) but superior improvement in nasal obstruction (55.95% vs. 39.18%, P < 0.05) and pharyngeal erythema (70.09% vs. 47.62%, P < 0.01) at Day 3 (Feng and Du, 2020). Another trial (n = 65) reported 88.33% clinical cure rates for influenza A-associated pharyngitis with XFOL, achieving viral clearance in 100% of per-protocol cases without severe adverse events (Zhou et al., 2020). Mechanistic studies attribute its effects to inhibition of NF-κB signaling and downregulation of pro-inflammatory cytokines (IL-6, TNF-α) (Jia et al., 2021). Despite promising results, the reliability of these conclusions is reduced due to the low methodological quality of the original studies. Moreover, existing studies lack data from positive-controlled trials. To address this evidence gap, we designed a non-inferiority randomized controlled trial and selected Xiao’er Yanbian Granules (XYG) as the positive control drug due to its proven efficacy in reducing tonsillar inflammation, inflammatory markers (IL-4, IL-1β, TNF-α, CRP), and symptom duration in pediatric pharyngotonsillitis (Wen et al., 2024). Using a non-inferiority margin of Δ = 0.1, based on previous non-inferiority clinical studies and the endorsement of clinical experts, this study will objectively validate XFOL’s effectiveness and safety in PAPT.

## Methods

### Ethical approval

This trial was approved by the ethics review committee of the Beijing University of Chinese Medicine Third Affiliated Hospital (ECPJ-BZYSY-2024-03, Beijing, China), and obtained ethical approval from each sub-center. All participants will sign a written informed consent form prior to enrollment. The development and reporting of the trial protocol adhere to the Consolidated Standards of Reporting Trials (CONSORT) 2010 Statement (Schulz et al., 2010). The trial has been registered in International Traditional Medicine Clinical Trial Registry (ITMCTR2024000569).

### Trial design

**FIGURE 1:**
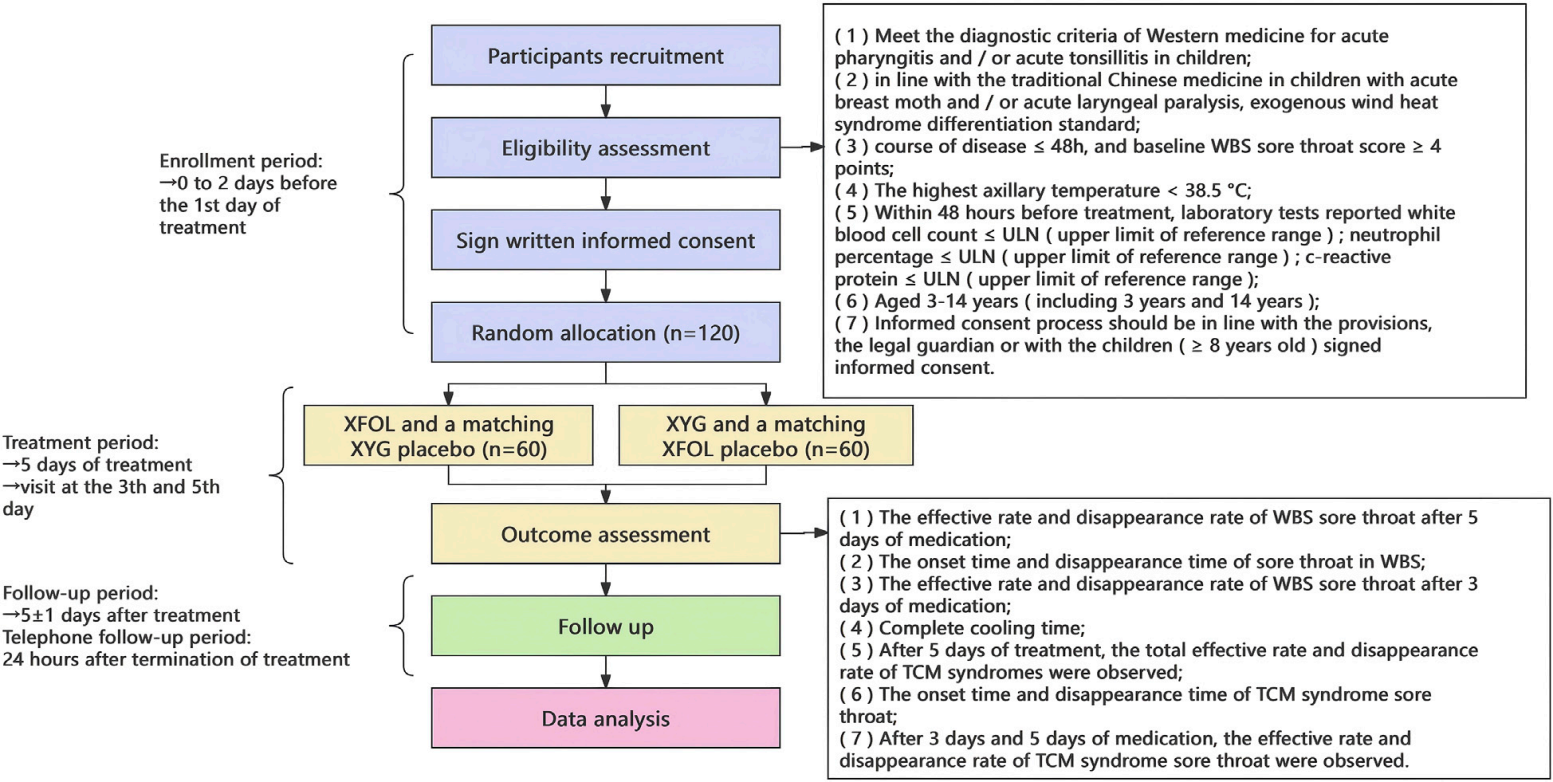
Flowchart of the trial design. XFOL, Xiao’er Fengreqing Oral Liquid; XYG, Xiao’er Yanbian Granules.

This trial employs a multi-center (three-centers), randomized, parallel-group, non-inferiority design. Eligible participants, after signing written informed consent, will be randomly assigned in an equal ratio to either the trial group or the control group. Enrolled participants will receive medication for a 5-day treatment duration. After completing 5 ± 1 day of continuous medication, participants will return to the hospital for follow-up, return the study medication and diary cards, and terminate treatment. The flowchart of the trial design is presented in Figure 1.

### Participant and public involvement

Suggested: No participants or members of the public were involved in the design or conduct of this trial.

### Study setting

The trial will be conducted at three medical centers in China (detailed in Table 1), with participant recruitment scheduled to begin on 28 October 2024. All patients will be recruited through hospital visits.

**TABLE 1:** Study setting details of trial.

No. of center	Hospitals	Location	Role of center	Anticipated participants
1	Beijing University of Chinese Medicine Third Affiliated Hospital	Beijing	Principle investigator	
2	Second Affiliated Hospital of Shandong University of Traditional Chinese Medicine	Jinan, Shandong	Collaborator	
3	Xiangyang Central Hospital	Xiangyang, Hubei	Collaborator	

### Diagnostic criteria

#### Standard medicine criteria

The diagnosis of acute pharyngitis and tonsillitis follows established guidelines (Chinese Association of Traditional Chinese Medicine, 2020; Hu, 2015; Liu and Gu, 2017; Zheng, 2002). Acute pharyngitis requires: (1) acute-onset sore throat with dysphagia; (2) pharyngeal mucosal hyperemia, swollen lymphoid follicles, or scattered purulent spots; (3) predisposing factors (e.g., viral/bacterial exposure, environmental irritants). Acute tonsillitis is defined by: (1) systemic symptoms (fever ≥38°C, chills, fatigue); (2) severe odynophagia with tonsillar erythema/swelling (Brodsky scale Grade I-III); (3) laboratory evidence (elevated WBC, neutrophils, or CRP for bacterial cases). Infants may present atypically with drooling, abdominal pain, or respiratory distress.

#### TCM syndrome differentiation

External wind-heat syndrome is diagnosed per TCM guidelines (Chinese Association of Traditional Chinese Medicine, 2020; Zheng, 2002), requiring:

Core features: (a) acute sore throat aggravated by swallowing; (b) erythematous pharynx/tonsils without suppuration.

Supporting signs: Fever (≥37.8°C), dry throat, yellow sputum, rapid floating pulse, red tongue with thin yellow coating.

Exclusion criteria: Purulent tonsils, thick greasy tongue coating, or deep pulse indicating heat-toxin or dampness patterns.

#### Diagnostic confirmation

The diagnosis is confirmed if the participant presents with one main symptom, one pharyngeal sign, at least two secondary symptoms, and the corresponding tongue and pulse signs.

#### Inclusion criteria

Eligible participants must satisfy all criteria:

Age 3–12 years, either sex.

Meeting the above diagnostic criteria for external wind heat syndrome.

Symptom onset within 48 h prior to enrollment.

No use of antipyretics, antibiotics, or antiviral drugs within 24 h before enrollment.

Legal guardian provides written informed consent and agrees to standardized follow-up.

#### Exclusion criteria

Participants are excluded if any of the following apply:

Alternative diagnoses: (1) Suspected bacterial infection (e.g., tonsillar exudate, cervical lymphadenitis, or fever ≥39.5°C). (2) Herpangina, hand-foot-mouth disease, or other viral exanthems.

Comorbidities: (1) Chronic pharyngitis/tonsillitis (>3 episodes/year). (2) Immunodeficiency, asthma, or severe cardiopulmonary diseases.

Medication factors: (1) Allergy to XFOL components or control drug (e.g., Lianhua Qingwen granules). (2) Use of immunosuppressants or systemic corticosteroids within 4 weeks.

Logistical contraindications: (1) Participation in other clinical trials within 30 days (2) Inability to comply with protocol (e.g., language barriers, geographic instability).

#### Study discontinuation

Participants may discontinue the trial under the following conditions (documented in Case Report Form, CRF):

Voluntary withdrawal: The participant or their legal guardian chooses to withdraw from the study at any time.

Protocol violations: Incorrect enrollment (e.g., retrospective diagnosis of bacterial infection), or non-adherence to medication (>20% doses missed).

Safety-related termination: (1) Severe adverse events (SAEs) requiring hospitalization. (2) Disease progression necessitating rescue antibiotics.

Investigator decision: (1) Clinical judgment deems discontinuation in the participant’s best interest. (2) Discontinued participants receive appropriate medical management per hospital protocols.

#### Sample size calculation

Primary endpoints: throat pain resolution rate and overall effective rate

Parameters: Expected throat pain resolution rate: 91.43% (intervention group) vs. 76.92% (control group), non-inferiority margin (Δ) = 0.1 (Zhu et al., 2014), one-sided α = 0.025, power (1-β) = 80% (Chow et al., 2017). Initial calculation using PASS 15 software yielded 34 participants per group.

Adjustments:

A 20% dropout rate adjustment (n = 34/0.8 ≈ 43) required 44 participants per group (total 88).

To account for potential challenges in pediatric compliance and ensure robust statistical power, the final sample size was increased to 120 participants (60 per group). Since non-inferiority must be demonstrated for both primary endpoints to consider the trial successful, no adjustment for multiple testing is required in the analysis phase.

#### Randomization, allocation concealment, and blinding

This multicenter trial will employ a central block randomization method (block size = 4) to ensure balanced allocation between the two groups, each comprising 60 participants. An independent statistician from the Center for Evidence-Based Chinese Medicine, Beijing University of Chinese Medicine, will generate the randomization sequence using SAS software, with site-specific coding ranges assigned to each participating center. Sequentially numbered, sealed, opaque envelopes will be used to maintain allocation concealment. Medications will be pre-packaged by personnel uninvolved in trial execution and stored in hospital pharmacies to preserve blinding.

Trained site investigators will enroll eligible participants and obtain informed consent. Upon enrollment, a designated third-party staff member will dispense study medications labeled with unique identifiers corresponding to the randomization list. To maintain blinding, both the participants and investigators will be unaware of treatment assignments throughout the trial. Blinding will also extend to outcome assessors and data analysts to minimize evaluation bias. All site personnel will undergo standardized training to ensure adherence to the trial protocol.

### Intervention

#### Trial group

Intervention: Participants in the trial group will receive XFOL and a matching placebo for XYG (4 g/sachet). Both the active drug and placebo are indistinguishable in appearance, taste, and odor. For ages 3–6 years, 5–10 mL of XFOL (or placebo), administered orally 4 times daily; for ages 6–14 years, 7.5–15 mL of XFOL (or placebo), administered orally 4 times daily. The oral solution and placebo must be shaken thoroughly before use. The color, smell and taste of the placebo for XYG drug are consistent with those of XYG.

XFOL ingredients: *Lonicera japonica*, Forsythia suspensa, Isatis indigotica, Mentha haplocalyx, Bupleurum chinense, Lophatherum gracile, Arctium lappa, Platycodon grandiflorus, Scutellaria baicalensis, Gardenia jasminoides, Phragmites communis, CaSO_4_·2H_2_O, Schizonepeta tenuifolia, Saposhnikovia divaricata, Bombyx batryticatus, Glycyrrhiza uralensis, Massa Medicata Fermentata, Citrus reticulata, Cryptotympana atrata, Paeonia lactiflora, Prunus armeniaca.

#### Control group

Intervention: Participants in the control group will receive XYG (National Drug Approval Number: Z62020014; 4 g/sachet) and a matching placebo for XFOL (10 mL/bottle). Both the active drug and placebo are identical in appearance, taste, and odor. For ages 3–5 years, 1 sachet (4 g) of XYG (or placebo), administered orally 3 times daily; for ages 6–14 years, 2 sachets (8 g) of XYG (or placebo), administered orally 2–3 times daily. The color, smell and taste of the placebo for XFOL drug are consistent with those of XFOL.

XYG ingredients: Honeysuckle, Belamcanda rhizome, Tinospora pubescens root, Platycodon grandiflorus root, Scrophularia ningpoensis root, Ophiopogon japonicus root, Artificial Bovis Calculus, Borneol.

#### Treatment course

The treatment duration is 5 days (±1 day) as this period covers the critical progression phase of the disease. Participants will return for follow-up visits after completing the course to return unused medications and logcards. A post-treatment telephone follow-up will be conducted 24 h after discontinuation to assess body temperature, concomitant medications, and adverse events.

#### Rescue therapy

Acetaminophen oral suspension (brand name: Tylenol; 100 mL:3.2 g) will be administered as antipyretic rescue medication for participants with axillary temperature ≥38.2°C (Liu and Gu, 2017). For dosage, 1–3 years (12–15 kg): 3 mL per dose; 4–6 years (16–21 kg): 5 mL per dose; 7–9 years (22–27 kg): 8 mL per dose; 10–12 years (28–32 kg): 10 mL per dose. For frequency, repeat every 4–6 h if fever persists, with a maximum of 4 doses within 24 h. Persistent high fever unresponsive to rescue therapy requires immediate medical intervention.

During the trial, the use of antibiotics, antiviral agents, or other TCM with purported effectiveness for pediatric acute pharyngitis/tonsillitis is strictly prohibited. Additionally, non-protocol treatments such as infrared therapy, cupping, and acupuncture are disallowed throughout the study and follow-up periods. Acetaminophen is permitted for fever management (axillary temperature ≥38.2°C), and its dosage must adhere to the product instructions. All concomitant medications, including rescue therapies, must be meticulously documented in the CRF and original medical records, specifying the generic drug names, dosages, administration times, and reasons for use to ensure comprehensive data integrity and protocol adherence.

### Outcome measures

#### Pain measurement

**FIGURE 2:**
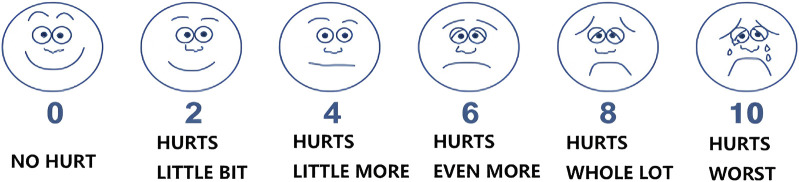
Faces Pain Scale for assessing throat pain in children.

The Wong-Baker Faces Pain Rating Scale (WBS) employs six facial expressions (0–10 scale) to quantify throat pain intensity (Figure 2). Children aged ≥3 years select the face best representing their current pain, with 0 indicating no pain (smiling face) and 10 reflecting severe pain (crying face) (Garra et al., 2010).

#### TCM symptom evaluation

The following table outlines the grading and quantification criteria for TCM syndrome symptoms, including primary symptoms (e.g., sore throat), secondary symptoms (e.g., dry throat, fever, cough), and physical signs (e.g., pharyngeal and tonsillar findings). Symptoms are scored on a severity scale (0–6 points for primary symptoms; 0–6 points for secondary symptoms and physical signs), with higher scores indicating greater severity. Tongue and pulse characteristics are recorded but not scored (Chinese Association of Traditional Chinese Medicine, 2020) (detailed in Table 2).

**TABLE 2:** Grading and quantification criteria for TCM syndrome symptoms.

Primary symptoms	Normal (0 points)	Mild (2 points)	Moderate (4 points)	Severe (6 points)
Sore throat (pain on swallowing)	None	Mild pain during swallowing	Pain during swallowing	Severe pain with difficulty swallowing

Secondary symptoms	Normal (0 points)	Mild (2 points)	Moderate (4 points)	Severe (6 points)
Dry throat	None	Mild discomfort	Dryness with burning or mild pain	Severe burning or pain
Fever	Axillary temperature ≤37.2°C in past 24 h	Axillary temperature 37.3°C–37.9°C	Axillary temperature 38°C–38.5°C	Axillary temperature >38.5°C
Cough with yellow sputum	None	Occasional cough, small amount of sticky yellow sputum	Intermittent cough, does not affect rest or sleep, sticky yellow sputum, easy to expectorate	Frequent cough affecting rest or sleep, sticky yellow sputum, difficult to expectorate

Physical signs	Normal (0 points)	Mild (2 points)	Moderate (4 points)	Severe (6 points)
Pharynx	None	Hyperemia of posterior pharyngeal wall	Hyperemia and edema of posterior pharyngeal wall	Significant hyperemia and edema of posterior pharyngeal wall, uvula, and lateral bands, with secretions
Tonsils	None	Tonsillar hyperemia	Tonsillar hyperemia and edema, with secretions or purulent spots	Tonsillar hyperemia and edema, with abundant secretions or purulent coating
Lymphadenopathy	None	Submandibular or cervical lymph nodes <1 cm, mild tenderness	Submandibular or cervical lymph nodes 1–2 cm, significant tenderness	Submandibular or cervical lymph nodes >2 cm, severe tenderness
Tongue and pulse	Tongue and pulse characteristics: recorded but not scored			

#### Baseline data

Prior to treatment initiation, comprehensive baseline data will be collected, including demographic characteristics (sex, age, height, weight, ethnicity), medical history (disease duration, past medical conditions, allergies, pre-trial medications), and diagnostic indicators (symptoms such as sore throat and fever, physical signs including pharyngeal/tonsillar erythema, and laboratory results).

#### Primary outcomes

The primary effectiveness endpoint is the throat pain resolution rate and overall effective rate at Day 5, assessed using the WBS score. Participants will record pain severity daily in logcards, with scores ranging from 0 (no pain) to 10 (severe pain). Effectiveness is defined as a ≥50% reduction in WBS score from baseline, while resolution requires a score of 0. The trial will be considered successful only if non-inferiority is demonstrated for both primary endpoints simultaneously, with the test treatment non-inferior to the control treatment.

#### Secondary outcomes

1. Time to Onset and Resolution of WBS-Assessed Sore Throat

Participants will record throat pain severity using the WBS score in daily logcards at baseline and every 24 h post-treatment. Onset time is defined as the first time period during which the WBS score decreases by ≥ 1-grade from baseline, while resolution time refers to the interval when the score decreases to 0.

2. Effectiveness and Resolution Rates of WBS-Assessed Sore Throat at Day 3

Daily WBS scores will be analyzed to calculate the proportion of participants achieving ≥50% reduction (effectiveness) or complete resolution (score = 0) by Day 3.

3. Complete Fever Resolution Time

Participants will record axillary temperature three times daily (morning, noon, evening) until fever subsides. Complete resolution is defined as sustained temperature <37.2°C for ≥24 h.

4. TCM Syndrome Effectiveness and Resolution Rates at Day 5

Investigators will evaluate total TCM symptom scores (primary and secondary symptoms, physical signs) at baseline and Day 5. Effectiveness requires ≥50% score reduction, while resolution is defined as a score of 0.

5. Time to Onset and Resolution of TCM-Assessed Sore Throat

Participants will self-report TCM-specific sore throat severity in logcards every 24 h. Onset and resolution are determined using the same thresholds as WBS score (≥1-grade reduction and score = 0, respectively).

6. TCM-Based Effectiveness and Resolution Rates on Days 3 and 5

TCM sore throat scores from daily logcards will be analyzed to calculate effectiveness (≥50% reduction) and resolution (score = 0) rates at both timepoints.

7. TCM Symptom-Specific Effectiveness and Resolution at Day 5

Individual TCM symptoms (e.g., dry throat, cough, pharyngeal signs) will be assessed by investigators. Effectiveness and resolution rates for each symptom/parameter are calculated based on score reductions (≥50% or to 0) at Day 5.

The trial procedures are divided into three phases: baseline (−1 to 0 days), treatment/follow-up (5 ± 1 day after treatment initiation), and post-treatment telephone follow-up (24 h after discontinuation). Key assessments and interventions across these phases are summarized in Table 3.

**TABLE 3:** Grading and quantification criteria for TCM syndrome symptoms.

Item	Baseline (−1 to 0 days)	Treatment/follow-up (5 ± 1 day)	Telephone follow-up (24 h post-discontinuation)
Screening	×		
Informed consent	×		
Demographics	×		
Physical examination	×	×	
Body temperature	Record body temperature every 8 h		×
WBS	Record WBS score every 24 h		
TCM sore throat symptoms	Record TCM sore throat symptoms every 24 h		
TCM symptom grading scale	×	×	
Blood/urine routine tests^a^	×	×	
C-reactive protein^a^	×		
Electrocardiogram^a^	×	×	
Liver/kidney function tests^a^	×	×	
Logcard distribution/collection	×	×	
Drug dispensing/collection	×	×	
Concomitant medication records	×	×	×
Adverse event monitoring		×	×

^a^ Notes: Means laboratory test items. Baseline tests may be waived if recent (<48 h) results are available. Post-treatment tests require participant/guardian consent. Liver/kidney function tests are optional based on clinical necessity.

### Safety assessments

#### Safety evaluation metrics

Safety assessments encompass three components: (1) clinical adverse reactions, monitored continuously post-treatment; (2) vital signs (blood pressure, respiratory rate, body temperature, and heart rate), measured at baseline and treatment endpoint; (3) laboratory tests, including complete blood count (WBC, RBC, HGB, PLT, NEUT, NEUT%, LYM%), C-reactive protein, urinalysis (WBC, RBC, GLU, PRO), liver function (ALT, AST, ALP, GGT/γ-GT, TBIL), renal function (BUN, Cr), and electrocardiogram. Pre-existing laboratory results within 48 h prior to enrollment may exempt retesting. Post-treatment laboratory tests (after 5 ± 1 day of medication) will be performed with participant/guardian consent. Liver and kidney function tests are optional based on clinical necessity. The incidence of adverse reactions serves as the primary safety endpoint.

#### Adverse event (AE) management

Definition: AEs refer to any unfavorable medical occurrences post-treatment, regardless of causality. Severity is classified using the NCI-CTCAE v5.0 criteria: Grade 1 (mild), Grade 2 (moderate), Grade 3 (severe), Grade 4 (life-threatening), and Grade 5 (fatal) (U.S. Department of Health and Human Services, 2025). Management includes: (1) continuing treatment with observation for mild AEs; (2) discontinuing treatment without intervention for moderate AEs; or (3) halting treatment and administering rescue therapy for severe AEs. For serious AEs (SAEs), immediate trial termination, emergency unblinding (if required), and prompt reporting to sponsors and ethics committees are mandated. All AEs are documented with details on onset/resolution time, severity, interventions, and outcomes.

### Drug accountability and medication compliance

#### Drug inventory management

During each follow-up visit, investigators will count the remaining medication or empty packaging returned by participants and inquire about adherence to the prescribed regimen (e.g., missed doses, dosage deviations). This information will be documented in the CRF to evaluate compliance.

#### Compliance assessment

Medication adherence is defined as strict adherence to the prescribed dosing schedule without unauthorized use of additional therapies. Compliance will be calculated using the medication counting method, supplemented by participant interviews when necessary:

$$\text{Compliance Rate }\left(\%\right)=\left(\text{Total medication consumed }/\text{ Total medication prescribed}\right)\times 100\%$$

### Data management

#### Data entry and modification

Prior to data entry, stringent controls are implemented to identify and correct errors, with all modifications documented and retained. Data managers collaborate with the principal investigator to establish data range checks and logic validation rules, encompassing automated system checks and manual medical reviews. Post-entry corrections require approval by the site principal investigator, ensuring traceability of all system operations through audit trails.

#### Database setup and data verification

This trial utilizes an electronic Case Report Form (eCRF) and Electronic Data Capture (EDC) system, designed to align with the protocol. Data managers implement automated logic checks and validation rules, supported by SAS software for comprehensive data verification. This includes identifying missing data, ensuring values fall within predefined ranges, and validating logical consistency. Investigators or clinical research coordinators (CRCs) input source data into the EDC, followed by multi-tiered verification (investigator, CRC, and monitor) and electronic signatures. Discrepancies are resolved through iterative queries, with all modifications documented in audit trails to ensure data integrity and traceability.

#### Blind review and locking

Unresolved queries are addressed during a blind review by sponsors, investigators, and statisticians to finalize the statistical analysis plan. The database is locked after joint authorization and undergoes initial unblinding for analysis. Post-trial, all data and documentation are archived for ≥2 years, with prior sponsor notification required before disposal.

#### Statistical analysis data set

Full Analysis Set (FAS): Includes all randomized participants who received at least one dose of the study drug and provided post-baseline effectiveness data. Missing primary outcomes are imputed using the last observation carried forward (LOCF) method under the intention-to-treat (ITT) principle. FAS serves as the primary population for effectiveness analyses.

Per-Protocol Set (PPS): A subset of FAS comprising participants with protocol adherence, complete baseline data, no major protocol deviations, and measurable primary endpoints. PPS is used for sensitivity analyses of primary outcomes.

Safety Set (SS): Encompasses all participants who received ≥1 dose and had safety assessments. Adverse event rates are calculated using SS.

#### Statistical analysis principles

No interim analysis will be conducted in this study. Following comprehensive data verification, missing values will be addressed, and erroneous entries or logical inconsistencies will be corrected to ensure data quality and completeness prior to formal statistical analyses. All analyses will be performed by investigators and independent biostatisticians using SAS 9.4 (SAS Institute Inc., Cary, NC) or equivalent software, with analysts blinded to treatment allocation. Hypothesis testing will employ two-tailed significance tests (except for pre-specified non-inferiority comparisons), with results reported as 95% confidence intervals (CI) and a significance threshold of α = 0.05. Statistical uncertainties and variability will be rigorously quantified to support robust clinical interpretations.

#### Missing values

For effectiveness endpoints, missing values are addressed using the LOCF method for the FAS. LOCF assumes that the last observed value remains unchanged for subsequent missing timepoints, providing a conservative estimate of treatment effects. This approach is widely used in clinical trials to minimize bias from participant dropout and ensure the robustness of ITT analyses.

For safety endpoints, no imputation is applied; analyses rely solely on observed data to avoid overestimating or underestimating adverse event rates. This ensures a more accurate representation of safety outcomes.

#### Demographic and baseline characteristics

Demographic and clinical baseline characteristics will be summarized by treatment and control group. Categorical variables (e.g., sex, ethnicity, medical history) will be reported as frequency counts (n) and percentages (%), with denominators reflecting available data. Continuous variables (e.g., age, height, weight, symptom duration) will be presented as means ± standard deviations (SD) for normally distributed data or medians with interquartile ranges (IQR) for non-normally distributed data. Missing data counts will be explicitly noted for all variables.

The following baseline parameters will be analyzed:

1. Demographics: Age (years), sex (male/female %), height (cm), weight (kg), ethnicity.
2. Medical History: Disease duration (days), allergies, pre-trial medications, prior episodes of pharyngitis/tonsillitis.
3. Clinical Metrics: Baseline axillary temperature (°C), pharyngeal/tonsillar erythema severity (graded 0–3), laboratory values (WBC, CRP, ALT, AST, Scr).
4. TCM Syndrome Scores: Baseline scores for sore throat, dry throat, cough, and pharyngeal signs using the TCM symptom grading scale.

No formal hypothesis testing will be performed for baseline comparisons; results will be interpreted descriptively to evaluate randomization balance.

### Analysis of primary outcome

Non-inferiority will be evaluated using a predefined margin (Δ = 10%), with the lower bound of the one-sided 97.5% CI for the risk difference (trial-control) between groups compared against −Δ. If this lower bound exceeds −Δ, non-inferiority will be established. The sample size was calculated based on this non-inferiority hypothesis with a one-sided significance level of 0.025, maintaining consistency between design and analysis phases. Between-group comparisons will utilize Chi-square tests for categorical outcomes, supplemented by mixed-effects logistic regression adjusted for baseline covariates (age, baseline WBS score). To ensure consistency in statistical inference, both FAS and PPS analyses must demonstrate non-inferiority to confirm the robustness of conclusions. The trial will be considered successful only when non-inferiority is established for both primary endpoints; therefore, no adjustment for multiple testing is necessary as this represents a conservative approach requiring success on all endpoints. Should non-inferiority be established, superiority testing will be conducted as a hierarchical testing procedure by assessing whether the lower bound of the 95% CI exceeds zero. Results will be reported as proportions, risk differences, and adjusted odds ratios with 95% CIs, with forest plots used to visually represent the treatment effects and their confidence intervals relative to both the non-inferiority margin and zero.

### Analysis of secondary outcomes

#### Time-to-event endpoints

For time to onset/resolution of sore throat (WBS or TCM-assessed) and complete fever resolution time, survival analyses will be conducted using Kaplan-Meier curves to estimate cumulative event probabilities, with between-group comparisons via log-rank tests. Results will be reported as median event times and adjusted hazard ratios (HR) derived from Cox proportional hazards models, accounting for baseline severity and age. Participants lost to follow-up will be censored at their last recorded observation.

#### Repeated measures and binary outcomes

Effective/resolution rates at Days 3 and 5 (WBS or TCM-assessed) and TCM symptom-specific scores will be analyzed using generalized estimating equations (GEE) with an exchangeable correlation structure to address repeated measurements. Binary outcomes (e.g., resolution yes/no) will be modeled via log-binomial regression, reporting risk ratios (RR) and 95% CI. Continuous TCM scores will use linear mixed-effects models, adjusted for baseline values and study center.

#### Sensitivity and subgroup analyses

Analyses will be repeated in the per-protocol set to confirm robustness. Missing data will be handled via multiple imputations for sensitivity checks. Subgroup analyses will stratify by age (3–6 vs. 7–12 years) and baseline symptom severity (WBS score ≥7 vs. <7).

#### Safety outcomes and adverse events

Safety analyses will focus on AE incidence, categorized by severity (NCI-CTCAE v5.0 grades 1–5), system organ class, and causality (definite/probable/possible/unlikely related to treatment). AE rates will be summarized descriptively as frequencies (%) within each treatment group, with no formal statistical testing due to expected low event rates. SAEs will undergo detailed case-by-case assessment for potential treatment relationships, with timelines and outcomes documented. All safety data will be analyzed in the SS, comprising participants who received ≥1 dose of the study drug.

## Discussion

This trial aims to provide robust evidence on the effectiveness and safety of XFOL for pediatric acute pharyngitis/tonsillitis (external wind heat syndrome), addressing a critical gap in evidence-based TCM interventions for pediatric upper respiratory infections. By employing a multicenter, double-blind, positive-controlled non-inferiority design with standardized training across sites, the study ensures methodological rigor and generalizability. The integration of both subjective (WBS, TCM symptom scores) and objective outcomes (fever resolution time, laboratory parameters) balances participant-reported experiences with clinically measurable endpoints. Predefined statistical methods, including survival analysis and repeated measures models, enhance the reliability of conclusions.

This trial has several limitations. First, primary outcomes (e.g., WBS-assessed sore throat) rely on subjective participant-reported measures, potentially introducing bias. However, objective metrics such as fever resolution time and laboratory-confirmed inflammatory markers (e.g., CRP) are incorporated to balance subjective assessments. Additionally, though non-inferiority tests can be used to confirm XFOL efficacy relative to the comparator, it is also necessary to conduct an analysis of the advantages and disadvantages of the XFOL. Compared with the positive control drug XYG, the advantage of XFOL lies in its composition, which enables it to comprehensively exert the effects of clearing heat and dispersing wind, as well as to reduce swelling and fever, relieve sore throat, and improve tonsil enlargement. On the other hand, its limitations include relatively weak efficacy for other types of throat diseases and limited research data on its long-term efficacy and safety. Despite these limitations, the trial’s multicenter design, standardized training across sites, and predefined statistical analysis plan enhance methodological rigor. Results will provide critical evidence for integrating TCM into pediatric care, addressing unmet needs in acute upper respiratory infection management.

Our study makes contribution to supplement current international reporting in the following three aspects. Firstly, it establishes a more scientific and rigorous scientifically rigorous statistical analysis framework for international reports, especially in the context of traditional Chinese medicine clinical trials, by providing solutions for reasonable sample size estimation, analysis methods for primary and secondary endpoints, and handling of missing data. Subsequently, this study establishes a novel hybrid evaluation model by integrating an efficacy framework that combines TCM syndrome scoring with pediatric-specific tools including the Wong-Baker FACES scale and age-stratified dosing. Finally, our study will supply extensive data and analysis report on the safety of XFOL in treating pediatric acute pharyngitis/tonsillitis.

## Conclusion

In summary, this multi-center, randomized, double-blind, positive-controlled, non-inferiority trial will evaluate the effectiveness and safety of XFOL for pediatric acute pharyngitis/tonsillitis. By implementing a pre-registered statistical analysis plan, the study will ensure methodological transparency and minimize the risk of outcome reporting bias, providing a robust framework for assessing Fengreqing Oral Liquid. Predefining endpoints, analysis populations, and non-inferiority criteria will safeguard against data-driven conclusions and enhance reproducibility.

We argue that this study will significantly contribute to the evidence-based integration of TCM into pediatric care.
